# Bioinformatics roadmap for therapy selection in cancer genomics

**DOI:** 10.1002/1878-0261.13286

**Published:** 2022-08-20

**Authors:** María José Jiménez‐Santos, Santiago García‐Martín, Coral Fustero‐Torre, Tomás Di Domenico, Gonzalo Gómez‐López, Fátima Al‐Shahrour

**Affiliations:** ^1^ Bioinformatics Unit Spanish National Cancer Research Centre (CNIO) Madrid Spain

**Keywords:** bioinformatics, drug prioritisation, next‐generation sequencing, precision oncology, treatment selection, tumour heterogeneity

## Abstract

Tumour heterogeneity is one of the main characteristics of cancer and can be categorised into inter‐ or intratumour heterogeneity. This heterogeneity has been revealed as one of the key causes of treatment failure and relapse. Precision oncology is an emerging field that seeks to design tailored treatments for each cancer patient according to epidemiological, clinical and omics data. This discipline relies on bioinformatics tools designed to compute scores to prioritise available drugs, with the aim of helping clinicians in treatment selection. In this review, we describe the current approaches for therapy selection depending on which type of tumour heterogeneity is being targeted and the available next‐generation sequencing data. We cover intertumour heterogeneity studies and individual treatment selection using genomics variants, expression data or multi‐omics strategies. We also describe intratumour dissection through clonal inference and single‐cell transcriptomics, in each case providing bioinformatics tools for tailored treatment selection. Finally, we discuss how these therapy selection workflows could be integrated into the clinical practice.

## Graphical abstract

Intertumour heterogeneity and intratumour heterogeneity have been revealed as key causes of treatment failure and relapse. This review aims to provide a bioinformatics roadmap and general guidelines to propose anticancer data‐driven treatment strategies for bulk and single‐cell omics data in order to target tumour heterogeneity. Moreover, we discuss how these workflows could be integrated into the clinical practice.

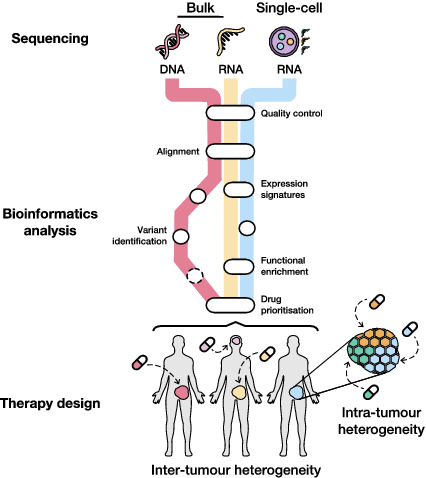

**Abbreviations**
ADR: adverse drug reaction
CNV: copy‐number variation
COSMIC: Catalogue Of Somatic Mutations in Cancer
DGE: differential gene expression
DNA‐seq: DNA sequencing
FCS: functional class scoring
FDA: Food and Drug Administration
GATK: Genome Analysis Toolkit
ICGC: International Cancer Genome Consortium
Indel: small insertions and deletions
ITH: intratumour heterogeneity
MTB: Molecular Tumour Board
NGS: next‐generation sequencing
ORA: over‐representation analysis
PCAWG: Pan‐Cancer Analysis of Whole Genomes
QC: quality control
RNA‐seq: RNA sequencing
scRNA‐seq: single‐cell RNA sequencing
SNV: single nucleotide variant
ST: spatial transcriptomics
SV: structural variant
sWGS: shallow whole‐genome sequencing
TCGA: The Cancer Genome Atlas
TMB: tumour mutational burden
TME: tumour microenvironment
UMAP: Uniform Manifold Approximation and Projection
VCF: variant calling file
WES: whole‐exome sequencing
WGS: whole‐genome sequencing

## 1 Introduction

Over the past few years, our understanding of cancer disease has enabled advances in diagnosis and treatment, contributing to improving survival rates in many tumour types. Current therapeutic management of primary and disseminated tumours includes surgical resection, radiotherapy, hormonal therapy, chemotherapy, targeted therapies and immunotherapy. Targeted therapies are considered a cornerstone of precision oncology, that is the use of cancer genomic information as a means to stratify individual patients for the administration of optimal therapeutic modalities [1, 2]. Targeted therapies have been conceived on the basis of the druggable genome paradigm (Box 1), that is the genes and gene products known (or predicted) to interact with available compounds [3]. In the recent years, efforts have been focused on defining new predictive biomarkers of anticancer drug efficacy, and as a consequence, the number of predictive biomarkers approved by the Food and Drug Administration (FDA) has increased from 39 in 2013 to 214 in 2022 (i.e. greater than fivefold in the last 10 years) [4]. Common examples of targeted therapies are the use of *BRAF* V600E inhibitors in melanoma patients, imatinib to target *BCR‐ABL* translocations in chronic myeloid leukaemia and PD1/PD‐L1 inhibitors for the immunotherapeutic treatment of melanoma, lung, renal and other cancer types. In addition, next‐generation sequencing (NGS) technologies have driven the discovery and development of new pharmacogenetic biomarkers, which play crucial roles in identifying drug responders and nonresponders, avoiding adverse effects and optimising drug dosage. Nevertheless, targeted therapy development is challenging since most of the druggable genome remains unstudied and the clinical setting of targeted therapies is still underdeveloped. Moreover, even with the consideration of genomic and transcriptomic patients' profiles, some patients may not respond to a genomically guided treatment. Furthermore, a prominent caveat of current targeted therapies is the onset of acquired resistance and thus clinical relapse, despite favourable initial responses in advanced disease [5, 6].

Box 1 **Druggable genome**

The druggable genome is formed by the set of genes encoding proteins that are or potentially can be targeted by drugs. Of the ∼ 20 000 coding genes present in the human genome, ∼ 3000 have been estimated to be druggable and less than 700 are currently targeted by FDA‐approved drugs [223].

Tumour heterogeneity has been revealed as a novel key factor in the failure of anticancer therapies. The findings provided by large‐scale cancer genomics projects such as The Cancer Genome Atlas (TCGA), the International Cancer Genome Consortium (ICGC) and the Pan‐Cancer Analysis of Whole Genomes (PCAWG) consortia [7, 8, 9] have clearly revealed a high multidimensional genomic heterogeneity among different tumour types but also within the same patient, thus underlining the idea that cancers are not single diseases but rather an array of disorders with distinct molecular mechanisms [10]. The concept of tumour heterogeneity encompasses both inter‐ and intratumour heterogeneity (ITH). The former refers to the existence of different genomic alterations among cancer patients or within the same individual (i.e. primary vs metastatic tumour), while the latter describes the intrinsic clonal diversity found within tumours occurring as a consequence of cancer somatic evolution and natural selection. Tumour heterogeneity has been related to different treatment responses [11, 12], the appearance of drug resistance [13, 14] and therefore the patients' clinical outcome [15, 16]. In order to reveal the relationships between ITH and clinical outcome, the TRAcking Cancer Evolution through therapy (Rx) (TRACERx) initiative is performing an extensive multi‐omics profiling of ITH in NSCLC, melanoma, prostate and renal cancer [17]. Deceased patients are corecruited to the Posthumous Evaluation of Advanced Cancer Environment (PEACE) (NCT03004755) study, which allows for metastatic sampling from multiple tumour sites. The Glioma Longitudinal Analysis Consortium (GLASS) is another international effort whose goal is the molecular characterisation of gliomas over several time points in order to understand tumour evolution and identify therapeutic vulnerabilities [18]. The characterisation of ITH has also benefited from single‐cell techniques that have allowed high‐resolution dissection of both tumour and tumour microenvironment (TME) cell composition. In this sense, the Human Tumour Atlas Network (HTAN) and other initiatives [19, 20] are generating single‐cell three‐dimensional atlases of tumour transitions, ITH and TME landscapes for a diverse set of tumour types. Undoubtedly, these valuable efforts have the potential to improve cancer detection, prevention and therapeutic discovery. However, the ITH, the tumour evolution and the potential for competitive release of resistant tumour subclones are not yet addressed in cancer therapeutics and clinical practice [21].

Bioinformatics provides a vast catalogue of methodologies and databases required to analyse, integrate and interpret cancer multi‐omics data. Remarkably, *in silico* drug prioritisation approaches (Box 2) have recently emerged to evaluate tumours' specific genomic alterations and transcriptomic profiles, matching them with tailored candidate treatments [22, 23, 24, 25, 26]. This review aims to provide a bioinformatics roadmap and general guidelines to propose anticancer data‐driven treatment strategies for bulk and single‐cell omics data covering both cancer research and precision oncology scenarios. Computational approaches required to generate tumour genomic and transcriptomic profiles and explore a tumour's functional activity are also discussed. Current algorithms for characterising tumour heterogeneity and dissecting ITH from multi‐omics sources will be addressed together with cutting‐edge methods that exploit the drug sensitivity of tumour cell subpopulations. Finally, the current limitations and perspectives in the development and improvement of novel computational approaches for precision medicine‐based therapies will also be discussed.

Box 2 ***In silico* prioritisation**

Precision medicine aims to make tailored prescriptions based on individual omics data. In order to do so, epidemiological, clinical and response data from previous patients are required. Drug prioritisation methods can integrate these sources of data and compute scores to rank the available treatments based on the predicted efficacy. These bioinformatics tools provide clinicians with evidence‐based guidance to prescribe the drug that better matches the characteristics of each patient.

## 2 Genomics‐based drug selection

NGS has been widely adopted for the analysis of tumour DNA extracted from clinical and biological samples with the aim of detecting clinically relevant genomic alterations for cancer diagnosis and treatment guidance. This section describes the computational workflow to analyse, detect and interpret DNA alterations (Box 3) (i.e. short variants and structural variants) that can guide cancer therapy selection using data generated by targeted, whole‐exome (WES) and whole‐genome sequencing (WGS) experiments. Cancer somatic mutations are the main focus of bioinformatics analyses aimed at identifying important druggable alterations, since targeted therapies directed against these variants would less likely affect healthy cells. However, germinal variants that affect drug substrates or metabolising enzymes can play an important role in drug effectiveness and toxicity and should not be overlooked when designing tailored treatment strategies.

Box 3 **Genomic variants**

According to their extent, genomic alterations can be classified into short or structural variants (SVs). Short variants can be subdivided into single nucleotide variants (SNVs) or small insertions and deletions of < 50 bp (indels). On the contrary, SVs affect genomic regions of ≥ 50 bp and can be further classified depending on whether the genetic material is conserved or lost. Balanced SVs arise as a consequence of inversions and translocations, whereas unbalanced SVs, also known as copy‐number variations (CNVs), are due to big insertions, deletions or duplications.

Genomic variants can also be classified as germline or somatic, depending on their origin and extent of the affected tissue. While germline mutations are inherited by the progeny and affect the whole organism, somatic mutations arise spontaneously and are localised in a specific tissue [224].

### 2.1 Short variants to guide therapies

**Fig. 1:**
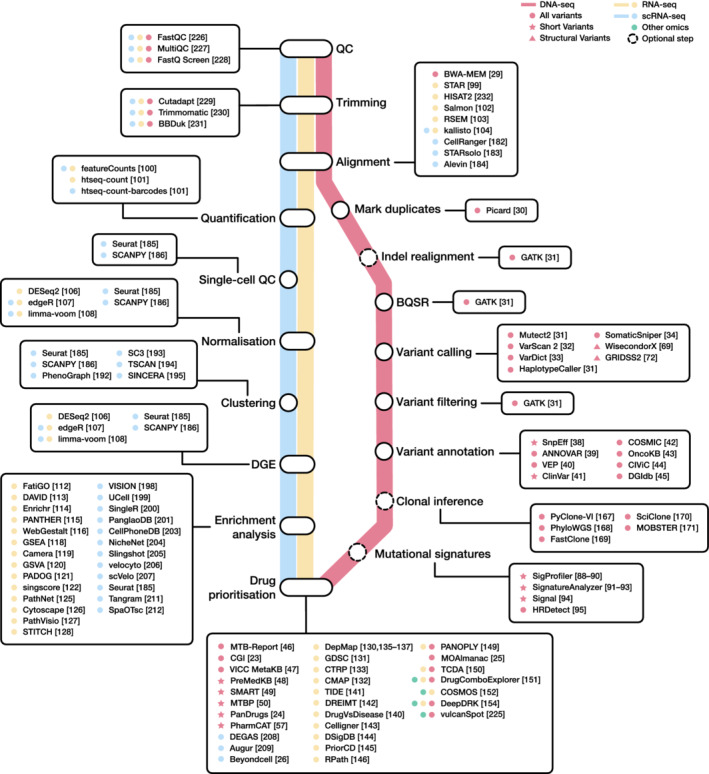
Roadmap for drug prioritisation from different omics profiles. The roadmap is represented as an underground map in which each next‐generation sequencing (NGS) technique is a different line and each step in the workflow is a station. Common steps between workflows are displayed as interchange stations. The names of the available tools are preceded by coloured symbols that indicate in which technique they can be applied. BQSR, base quality score recalibration; DGE, differential gene expression; QC, quality control.

The first step for genomics‐based drug selection is to identify clinically relevant alterations in cancer patients via a variant calling analysis. According to GATK Best Practices [27], the general workflow of variant calling consists of nine steps: quality control (QC) and trimming, alignment, marking duplicates, local realignment of indels, base quality score recalibration (BQSR), variant calling, filtering and annotation of variants [28] (Fig. 1). Briefly, after performing sample QC and trimming, the raw reads are aligned to the reference genome with tools such as BWA‐MEM [29]. Then, duplicated reads must be removed with Picard [30]. In order to reduce alignment artefacts and obtain more accurate sequencing quality estimations, further processing can be done using GATK tools [31]. There is a broad selection of variant calling tools such as Mutect2 and HaplotypeCaller [31], VarScan 2 [32], VarDict [33] or SomaticSniper [34] that can be used to identify short variants, which are comprised of single nucleotide variants (SNVs) and insertions or deletions (indels) of less than 50 base pairs (bp). The reported variants must be filtered in order to remove low‐quality calls and subsequently annotated with information about their biological impact, their frequency in the population and their clinical relevance. This type of analysis mainly focuses on somatic variants occurring in coding regions. Nonsynonymous SNVs are considered as more damaging, since they alter the final sequence of the encoded protein and might affect its correct folding and function [35]. Furthermore, somatic genomic alterations can be classified according to their frequency in the population as rare variants or polymorphisms, which are considered clinically benign due to their high frequency (> 1%). In most patients, at least one detected somatic alteration is potentially clinically relevant [36, 37] since it either changes the gene function, suggests the use of surveillance measures for prevention or early detection, helps to establish a diagnosis, influences the prognosis or guides the selection of therapies.

Some tools for automatic variant annotation are SnpEff [38], which determines the biological impact of candidate variants; or ANNOVAR [39] and the Variant Effect Predictor (VEP) [40], which additionally provide information about the frequency of each variant in the population. On top of that, there are many public data repositories of acquired knowledge about variants, drugs and their interconnections that are useful to annotate candidate somatic variants with. Some examples are ClinVar [41], a database of genetic variants and their clinical repercussions; the Catalogue Of Somatic Mutations In Cancer (COSMIC) [42], a knowledgebase with information about the impact of somatic variations in cancer; OncoKB [43] and CIViC [44], two resources that link somatic cancer variants with their clinical and therapeutic implications; or DGIdb [45], a database of gene–drug associations.

Furthermore, a number of methodologies and bioinformatics tools have been developed with the objective of making cancer variant interpretation easier and suggest possible treatments based on previous evidence (Table 1). Most of these resources are patient‐centred, require the somatic variants from the tumour and can be classified depending on the nature of the input data. If a list of variants is available, resources such as MTB‐Report [46], the Cancer Genome Interpreter (CGI) [23], the Variant Interpretation for Cancer Consortium meta‐knowledgebase (VICC metaKB) [47], PremedKB [48] or the SMART Cancer Navigator [49] can be useful. Some of these tools accept not only a list of short variants, but also disease and/or drug queries as input. In case a variant calling file (VCF) is available, the user can opt for MTBP [50] or PanDrugs [24]. The latter accepts both types of inputs and drug and gene queries. There have also been other approaches to guide therapy prescription on a large scale in order to obtain a general view and observe trends in cohorts of different tumour types [51].

**Table 1:** Bioinformatics tools for genomics‐based drug prioritisation.

Name	Description	Input	Output	URL
MTB‐Report [46]	R script that filters and classifies cancer variants into levels of evidence using gene‐drug databases	Tables with SNVs, CNVs and gene fusions (somatic)	Molecular Tumour Board (MTB) report with actionable variants in PDF	https://github.com/jperera‐bel/MTB‐Report
Cancer Genome Interpreter (CGI) [23]	Web tool that annotates cancer variants and identifies potential oncogenic alterations and genomic biomarkers of drug response	List of SNVs, indels, CNVs and/or gene fusions (somatic)	Downloadable tables of (a) annotated variants, including information about the oncogenicity and biological consequence, and (b) drug–variant associations with evidence level and response prediction	https://www.cancergenomeinterpreter.org/home
VICC MetaKB [47]	Web tool for cancer variant interpretation that harmonises six different variant annotation knowledgebases with information about variant, gene, disease and drug associations and their corresponding evidence levels	List of variants (somatic), including gene fusions, genes, diseases and/or drugs	Interactive report with variant–gene–disease–drug associations, each one with its evidence label and supporting links	https://search.cancervariants.org/#*
PreMedKB [48]	Web tool for integrating information on diseases, genes, variants, drugs and the relationships between any two or more of these four components	List of short variants (somatic), genes, drugs and/or diseases	Interactive semantic network displaying components as nodes and their relationships as edges. Results can be downloaded in either JSON or PNG format	http://www.fudan‐pgx.org/premedkb/index.html#/home
SMART Cancer Navigator [49]	Web application for variant interpretation that associates the corresponding genes to diseases, known drugs and relevant clinical trials	List of short variants (somatic and germline)	Interactive report with variant, gene, disease and drug information	https://smart‐cancer‐navigator.github.io/home
PanDrugs [24]	Web tool to prioritise anticancer drug treatments according to individual genomics data. pandrugs computes two scores, the Gene Score (GScore) and the Drug Score (DScore). The GScore ranges from 0 to 1 and is estimated according to gene essentiality and tumoral vulnerability, gene relevance in cancer, the biological impact of mutations, the frequency of gene alterations and their clinical implications. The DScore ranges from −1 to 1, considers drug indication and status, gene–drug associations and number of hits and estimates resistance (negative values) or sensitivity (positive values)	VCF, a list or a ranking of genes or a drug query (somatic)	Report with a prioritised list of anticancer therapies. pandrugs resolves the Best Therapeutic Candidates based on the accumulated and weighted scoring of the GScore and the DScore	https://www.pandrugs.org/#!/
MTBP [50]	Web tool that annotates somatic and germline short variants (SNVs and indels) functionally and clinically, categorising the cancer biomarkers (diagnosis, prognosis and drug response) found in the tumour	VCF or a list of short variants (somatic and germline)	HTML report with annotated variants, the evidence supporting the variants' functional classification and their associated actionability	https://mtbp.org/
PharmCAT [57]	A tool for identifying germinal variants, inferring patient's haplotypes and diplotypes and suggesting treatments following the Clinical Pharmacogenetics Implementation Consortium (CPIC) guidelines	VCF (germline)	HTML/JSON report with drug suggestions based on germinal variants	https://pharmcat.org/

Most of these approaches aim to prioritise drugs based only on somatic variants. However, germinal variants are also crucial in drug metabolism, and therefore in drug effectiveness and toxicity [52]. Thus, patients can have different responses to the same treatment, ranging from responsiveness to ineffectiveness or even adverse drug reactions (ADRs), which are important causes of morbidity and mortality and represent a source of financial burden to healthcare systems [53]. Differences in drug response are mainly due to genetic variation in genes encoding for drug substrates or genes that participate in the metabolism and transport of xenobiotics [54]. By assessing the germinal variants of each patient and mining pharmacogenomic databases such as DrugBank [55], PharmGKB [56], or the Table of Pharmacogenomic Biomarkers in Drug Labeling [4], effective compounds could be prioritised over ineffective or ADR‐causing drugs. PharmCAT [57] is a tool developed for suggesting tailored treatments based on germinal variants found in a VCF. Moreover, some resources such as the MTBP have been developed to account for both germinal and somatic variants.

Recent publications [58, 59] provide comprehensive lists of variant annotation knowledge bases and bioinformatics tools for variant interpretation, biomarker identification, drug prioritisation and response prediction. All these resources were conceived as supporting tools to inform clinicians of the available treatment options for their patients.

Interestingly, the identification of novel biomarkers of immunotherapy response has become one of the great challenges in oncology. Tumour mutational burden (TMB) has established itself as a promising genomic biomarker that may help identify patients who are most likely to benefit from immunotherapy in a wide range of tumour types [60]. TMB is calculated by counting the total number of somatic alterations divided by the total size in Mbp of the regions that have been sequenced. Nevertheless, there is a lack of standardisation for TMB assessment, which makes it difficult to use as a biomarker. High TMB is associated with improved or clinically relevant patient response to immunotherapy; however, the utility of this biomarker has not been fully demonstrated across all cancer types [61]. Moreover, using bioinformatics techniques, it is now possible to unravel the TMB content and generate *in silico* hypotheses beyond the TMB‐based stratification of patients. This way, we can prioritise and select targeted therapies based on the presence of mutations for which treatments already exist. This is the case of PanDrugs [24], a platform that prioritises drug treatments based on actionable mutations present in TMB.

### 2.2 Structural variants and mutational signatures to guide therapies

Genomic sequence alterations that affect large regions (≥ 50 bp) fall under the umbrella of what is known as structural variation (SV). A SV is composed of several types of events arising from different mutational mechanisms. Some of these events, such as deletions, insertions or duplications, result in changes in the amount of genomic sequence. These changes are known as copy‐number variations (CNVs) [62, 63, 64, 65]. Throughout history, a series of different techniques have been applied to study CNVs. The decreasing costs of WGS experiments combined with the constant improvement of variant calling methods are positioning WGS‐based CNV calling as the preferred technique for the analysis of CNV [66]. CNV can be studied through WGS experiments by detecting areas in the genome that have more or less reads than would be normally expected. This method is commonly known as depth of coverage (DOC) analysis. CNV has seen an increase in its applicability in the clinical diagnostics environment given its robustness to produce results with shallow levels of sequencing depth, usually defined as 0.1× to 1.0× coverage of the genome [67]. Even though most of the currently available CNV characterisation tools are aimed at the research environment [68], tools such as WisecondorX have been created with the specific goal of exploiting CNV calling using shallow WGS (sWGS) in the clinical setting [69]. An essential requirement for the study of mutational events is the ability to distinguish potentially significant events from those present in the healthy population [65]. Several approaches exist to achieve this, with the two main categories being those that do not require a normal reference, or reference‐free, and those that do [69]. Reference‐free approaches normalise the samples using known features of the human genome such as GC content and mappability. Reference‐based tools require as a reference either a single normal sample associated with the sample of interest or what is sometimes known as a Panel of Normals (PON) [70]. These normal samples have the goal of removing variation from the results, which could in fact be caused by experimental procedures (e.g. sample handling, preparation and sequencing equipment).

Structural variant events, whether CNV‐causing or not, can be extremely complex in terms of the changes they produce on the sequence of the genome [63, 71]. The characterisation of these events depends on methods such as the analysis of paired reads and split reads, and the *de novo* assembly of the genome of the sample of interest. The short nature of NGS reads imposes some limitations on these types of analyses [63]. Long‐read sequencing has given rise to a new generation of tools and approaches that aims at filling this gap in our ability to understand SVs [72]. Furthermore, unlike next‐generation machines, nanopore‐based sequencers offer great portability and the possibility of analysing data as it is generated (i.e. in a streaming fashion). Tools are already being developed with the aim of enabling the characterisation of SV for clinical diagnostics [73].

Structural variation has a potential impact on both germline and somatic genomic instability that affects disease development and might help to select therapies and report on patients' drug response. For instance, chromosomal translocations are relevant in the diagnosis of haematological malignancies but also lead to therapeutic approaches targeting fusion proteins such as BCR‐ABL1 in chronic myeloid leukaemia [74]. Some bioinformatics tools designed to prioritise drugs based on short variants also accept CNVs [23, 46] and/or gene fusions [47] as input. More advanced approaches for taking advantage of sWGS CNV calling for diagnostic purposes include efforts towards generating CNV‐based signatures, which may allow for more precise diagnostics and treatment selection [75]. Nevertheless, SVs are not yet commonly being used as molecular targets or biomarkers to guide patient‐specific treatment [76].

On the contrary, mutational signatures identified in genomic DNA can reveal unique patterns of mutational processes that occurred during the course of cancer development [77, 78]. These mutational signatures can include single base substitutions (SBS), doublet base substitutions (DBS), indels, CNV and genome rearrangements [79]. Interestingly, mutational signatures may be informative for guiding the identification of therapeutically targetable biomarkers, suggesting their application in personalised therapeutic approaches. In particular, several studies have found that tumours harbouring mutational signatures of DNA damage repair deficiency may show therapeutic responsiveness for either DNA damaging agents or immunotherapy [80, 81, 82]. For example, a mutational signature associated with pathogenic mutations in *BRCA1* and *BRCA2* genes has been identified in several cancer types, including breast and ovarian cancer, suggesting deficient homologous recombination (HR) and sensitivity to PARP inhibitors [83]. On the contrary, previous exposure to DNA‐damaging agents such as chemotherapy has been associated with drug resistance [84]. Interestingly, mutational signatures can be used as the mutational footprints of cancer therapies to estimate the contribution of different treatments to the TMB and can reveal their long‐term side effects in the genome [85].

There are several computational strategies for performing mutational signature analyses that in general differ on the mathematical properties of mutational signature discovery and can be grouped into two categories: methods that aim to discover novel signatures (*de novo*) or methods to detect already known and validated mutational signatures (refitting) [86, 87]. SigProfiler [88, 89, 90], a framework used for the previous version of COSMIC, and SignatureAnalyzer [91, 92, 93] were the two *de novo* tools used to analyse a large collection of cancer genomes from PCAWG, TCGA and ICGC projects [79]. It is worth noting Signal, a recently published web‐based tool for mutational signatures that also calculates the associations between gene drivers and mutational signatures that could provide novel therapeutic dependencies [94]. Moreover, HRDetect is a predictor of HR deficiency that can be useful to stratify patients based on their expected sensitivity to PARP inhibitors [95].

## 3 Transcriptomics‐based drug selection

Transcriptomics profiling has a wide range of applications in cancer research, from tumour classification, diagnosis and prognosis to therapeutic selection of drug candidates. Gene expression measures have already been incorporated in molecular diagnostics techniques such as the MammaPrint expression panel or the Oncotype DX Breast Recurrence Score to guide clinical decision‐making [96, 97].

This section focuses on bioinformatics approaches to prioritise therapeutic candidates based on gene expression (Fig. 1). First, we briefly summarise the most common steps in RNA sequencing (RNA‐seq) workflows. Next, we discuss functional enrichment approaches aimed at revealing biological patterns underlying gene expression. Finally, we review bioinformatics strategies for transcriptome‐based drug prioritisation depending on the data format and type.

RNA‐seq has become the most used NGS technique to detect and quantify the presence of RNA in biological samples. One of the first steps in a standard RNA‐seq data analysis is to generate a matrix of un‐normalised gene counts by aligning raw sequence reads to a reference genome or transcriptome [98]. The STAR aligner [99], and aggregation packages such as featureCounts [100] or htseq‐count [101] are some of the most widely employed tools to achieve the above step. Alternatively, alignment‐free methods such as Salmon [102], RSEM [103] or kallisto [104] output transcript‐level estimates, are then summarised to the gene level with R packages such as tximeta [105]. Next, the raw gene expression matrix must be normalised and transformed to stabilise intersample variance. Afterwards, differential gene expression (DGE) analysis extracts significant differences in RNA abundance between experimental conditions. Well‐established methodologies such as DESeq2 [106], edgeR [107] or limma‐voom [108] perform both normalisation and DGE in tandem.

Functional enrichment is often performed following a DGE analysis with the aim of revealing biological relationships in the differentially expressed genes list and of identifying underlying coordinated patterns (e.g. functional pathways and regulatory modules) in the expression matrix. Functional enrichment methods can be categorised into three main types: (a) over‐representation analysis (ORA); (b) functional class scoring (FCS); and (c) pathway topology [109]. They commonly exploit annotations from public databases, ontologies or related gene terms (gene sets) based on their involvement in a pathway, biological function or specific cellular compartment [110, 111]. ORA methods statistically evaluate the proportion of genes that share a particular annotation in a gene list of interest with respect to what is expected by chance. Web tools such as FatiGO [112], DAVID [113], Enrichr [114], PANTHER [115], WebGestalt [116] and others [117] follow this approach. As an alternative to ORA methods, FCS methods consider that coordinated changes in functionally related genes are as important as large expression changes in individual genes. To this end, FCS tools rely on ranked lists (i.e. gene expression rankings) to generate a single pathway‐level enrichment score, which is tested for statistical significance. Widely employed methods include GSEA [118], Camera [119], GSVA [120], PADOG [121], singscore [122] and others [123]. Finally, pathway topology analysis adds another information layer by taking into account gene–gene interactions along with gene‐level statistics to identify regulatory changes in pathways. Pathway topology methods have been extensively reviewed by Ihnatova et al. [124]. Remarkable contributions include PathNet [125], which leverages the connectivity between genes of the same pathway along with the differences in gene expression between conditions. Cytoscape [126] and PathVisio [127] offer powerful visualisation and analysis tools tailored to biological interaction networks. Moreover, STITCH [128] integrates information from metabolic pathways, compound structures and drug–target relationships to generate a network of compound–protein interactions.

Drug prioritisation methods can employ transcriptomic profiles as input to suggest which treatments will be most effective for a given tumour sample. Multiple bioinformatics strategies are available depending on the nature and complexity of the data source, ranging from individual genes (e.g. a single overexpressed gene from a DGE analysis) to whole expression data matrices.

The integration of genomic and transcriptional profiles together with drug response profiles has allowed the advancement of drug repositioning and drug combination predictions [129]. By finding its druggable weak spots, pharmacogenomics studies (Box 4) have been demonstrated to be useful in aiding treatment selection in cancer cell lines [130, 131, 132, 133, 134, 135, 136, 137]. Resources and tools such as the DepMap [130, 135, 136, 137], the GDSC [131] and the CTRP [133] are remarkable efforts for drug prioritisation using transcriptomics. In all cases, the input is a single gene that can be queried against large databases of pharmacogenomics assays, allowing researchers to correlate the expression level of a gene of interest with the susceptibility to drugs in thousands of cancer cell lines.

Box 4 **High‐throughput drug screenings**

High‐throughput screenings are assays in which large libraries of compounds are tested in order to discover candidate drugs with activity against a target.

Gene expression signatures (a list of genes whose expression is associated with a given condition) can be interrogated for drug prioritisation applying the signature reversal approach, which relies on the fact that the expression pattern of drugs indicated for a disease is often negatively correlated with the changes in gene expression induced by that disease [138]. For instance, Cheng and colleagues mined TCGA to generate an expression signature of *EGFR* activity, which they associated with tumour sensitivity to EGFR inhibitors and other tyrosine kinase inhibitors [139]. Similarly, the Connectivity Map (CMAP) [132] project is generating a comprehensive catalogue of cellular signatures representing systematic perturbation to pharmacologic and genetic perturbagens. Researchers can freely access the CMAP database or interrogate signatures of interest through its Web portal (Table 2). DrugVsDisease mines microarray databases to generate ranked expression profiles for the comparison of drug and disease gene expression profiles [140]. It features a precalculated ranked list of differentially expressed genes for 1309 drug compounds applied to cancer cell lines readily available for signature reversal. Expression signatures have also been used to predict response and prioritise compounds for immunotherapy. TIDE evaluates biomarkers to predict immune check blockade clinical response for patient stratification [141]. DREIMT performs drug prioritisation analysis for immunomodulation suggesting candidate immunomodulatory drugs targeting user‐supplied gene expression signatures [142].

**Table 2:** Bioinformatics tools for transcriptomics‐based drug prioritisation.

Name	Description	Input	Output	URL
The Connectivity Map (CMAP) [132]	Catalogue of gene expression signatures representing systematic perturbations with genetic and pharmacologic perturbagens. Features a Python library for programmatic access and cloud powered tools for quick interrogation of signatures, genes and compounds	Multiple inputs, depending on the Web tool	Multiple outputs, depending on the Web tool	https://clue.io/
The Cancer Dependency Map (DepMap)[130, 135, 136, 137]	Systematic study with the aim of uncovering genetic dependencies, small molecule sensitivities and discovering the biomarkers that predict them. Web portal powered by freely available multi‐omics data sets	Single gene, compound, cell line or lineage as plain text	Scatterplots, linear models and correlations between features	https://depmap.org/portal/
DREIMT [142]	Web tool/RESTful API for hypothesis generation and prioritisation of drugs capable of modulating immune cell activity from transcriptomics data	Gene list as plain text or as a comma‐separated file	Prioritised list of drug candidates for immunomodulation	http://dreimt.org/
DSigDB [144]	Gene set collection of annotated compounds and drugs	None	None	http://dsigdb.tanlab.org/DSigDBv1.0/
RPath [146]	Network‐based approach that proposes drugs for a disease by means of a knowledge graph and perturbation signatures	Knowledge graph with source, target and polarity as a tab‐separated file. Gene expression data as a dictionary of keys (genes) and values depending on the gene expression	Prioritised list of drug candidates for a given disease	https://github.com/enveda/RPath
The Cancer Druggable Atlas [150]	Comprehensive catalogue of potential druggable genes across cancers. Publicly available through the Functional Cancer Genome data portal	HNGC symbol as plain text	Multi‐omics profile of the target gene and its predicted druggability	http://fcgportal.org/TCDA/
vulcanSpot [225]	Web tool/RESTful API which mines massive screenings data to identify genetic dependencies and prioritise therapeutic candidates using a combination of known drug–gene relationships and drug repositioning strategies	Single gene as plain text	Ranked list of genetic dependencies alongside therapeutic candidates	http://vulcanspot.org/
PANOPLY [149]	R package that uses machine learning and knowledge‐driven network analysis to identify and analyse patient‐specific alterations (CNVs, germline and somatic short variants, fusion transcripts, gene expression and expressed mutations) driving oncogenesis and prioritise drugs that target the networks and pathways associated with these alterations	CNVs, SNVs (somatic and germline), expressed SNVs and expressed genes	Integrated multi‐omics case report of the patient with prioritisation of anticancer drugs	http://kalarikrlab.org/Software/Panoply.html
COSMOS [152]	R package that leverages extensive prior knowledge of signalling pathways, metabolic networks and gene regulation with computational methods to estimate activities of transcription factors and kinases, as well as network‐level causal reasoning	At least two sources of information from human transcriptomics, phosphoproteomics, metabolomics or fluxomics assays	Integrated trans‐omics network of estimated activities of kinases and transcription factors	https://saezlab.github.io/cosmosR/
CellPhoneDB [203]	Publicly available repository of curated receptors, ligands and their interactions	Counts data (either a TXT, H5AD or H5 file) or a path to the folder containing a 10× output with mtx/barcode/features files	Multiple comma‐separated files detailing ligand–receptor interactions along with statistical significance metrics for each pair	https://www.cellphonedb.org/
NicheNet [204]	R package that predicts ligand–receptor interactions between sender and target cell subpopulations that might drive gene expression changes	NicheNet's tables with prior information about ligand–receptor pairs, a preprocessed scRNA‐seq matrix (or a Seurat object) and a list of genes in the target subpopulation whose expression might be influenced by cell‐to‐cell communication	Table with the probability of each ligand in the sender subpopulation of driving the expression changes of the target genes	https://github.com/saeyslab/nichenetr
Beyondcell [26]	R package for single‐cell‐based drug prioritisation	Preprocessed scRNA‐seq matrix (or a Seurat object)	Prioritised ranking of the differential sensitivity drugs between chosen conditions	https://bioinformatics.cnio.es/tools/
Augur [209]	R package for prioritisation of cell types based on the response to an experimental perturbation. Augur trains a machine learning model for each cell type and quantifies the separability of perturbed and nonperturbed cells. The most separable cell type is assumed to be the most responsive to the perturbation	Preprocessed scRNA‐seq object (either Seurat, Monocle 3 or SingleCellExperiment) containing metadata associated with each cell, including the cell type annotations and sample labels to be predicted	Rank of cell types according to the amount of responsiveness to the perturbation	https://github.com/neurorestore/Augur
DEGAS [208]	R package that implements a deep transfer learning framework for prioritising cells in relation to disease attributes (such as diagnosis, prognosis and response to therapy) retrieved from patients	scRNA‐seq and gene expression matrices, as well as patient metadata with clinical information	Single‐cell metadata with clinical annotations for each cell and/or patient metadata with cell compositions	https://github.com/tsteelejohnson91/DEGAS

The whole normalised expression data matrix can also be used to prioritise drugs. For instance, following the Celligner methodology [143], the transcriptomic profile of individual samples can be aligned to the most similar cancer cell line, allowing researchers to harness the extensive pharmacogenomics profiling of said models to draw hypotheses about drug susceptibility. Moreover, GSEA can be used in conjunction with DSigDB [144], a database of drug gene sets, to find whether an experimental condition is enriched in genes participating in a given drug response. On the contrary, single sample enrichment methods such as GSVA perform the aforementioned enrichment sample‐wise instead of per condition, transforming a gene matrix to a drug signature enrichment matrix. Then, this matrix can be used for clustering, applying linear models or other approaches.

Finally, network‐based algorithms leveraging pathway topology have also been used for drug prioritisation. PriorCD [145] makes use of a network propagation algorithm and a drug–drug similarity network, along with pathway activity profiles to prioritise candidate drugs in cancer. Similarly, RPath [146] relies on a knowledge graph built from disease, protein and drug causal relations along with disease and perturbed expression signatures to prioritise compounds for a given disease.

## 4 Integrative multi‐omics strategies for drug selection

High‐throughput technologies have opened up the possibility of integrating orthogonal omics layers for a more comprehensive understanding of biological systems [147]. Drug prioritisation could also benefit from such integration [148]. Methods such as panoply [149] or MOAlmanac [25] (Table 2) integrate genomic and transcriptomic data to identify and prioritise drug targets. The Cancer Druggable Gene Atlas (TCDA) [150] is a recently published database with information about genomic alterations including short variants, CNVs and gene fusions, expression, gene dependency and druggability. DrugComboExplorer [151] takes into account DNA sequencing, gene copy number, methylation and expression data from cancer patients to (a) identify driver signalling pathways and (b) propose anticancer drug combinations.

Transcriptomic networks can also be enriched with subsequent omics layers to provide functional insight transcending individual layers. COSMOS [152] integrates phosphoproteomics, transcriptomics and metabolomics to estimate the activity of kinases and transcription factors. Finally, deep learning algorithms are becoming promising approaches for multi‐omics integration thanks to their capability of capturing nonlinear and hierarchical features [153]. For instance, DeepDRK [154] leverages genomics, transcriptomics, epigenomics and chemical properties of compounds to predict drug susceptibility in both cancer cell lines and patients.

The application of bioinformatics methodologies to immunotherapy as part of precision oncology is still in its early stages. However, tools already exist that allow the design of personalised vaccines [155]. From the large lists of potential neoantigens generated from NGS, it is possible to select those with the highest probability of success, that is to find an optimal design to generate efficient vaccines based on patient‐specific neoantigen profiles. Neoantigen prediction pipelines such as pVACtools [156] include different computational tools to detect neoantigens from tumour DNA‐seq and RNA‐seq data. They also estimate the individual's HLA class and prioritise neoantigens based on the molecular match with the patient's MHC and other parameters [157]. Moreover, there are programs such as CIBERSORTX [158] or MCP‐counter [159] capable of inferring the presence of immune infiltrates in tissue from expression data. Knowledge of the type of immune infiltration present in a tumour might serve as a guide, together with TMB values, for treatment selection. Finally, it should be noted that most of the proposed methodologies are still far from being applied in the clinic, although some such as the prioritisation of drug treatments or neoantigens based on TMB content are beginning to be used in clinical trials [155].

## 5 Targeting tumour heterogeneity: ith and drug selection

ITH functional diversity within individual tumours has been related to somatic SNVs, SVs, transcriptomic and epigenetic changes influencing gene expression levels, the TME status and the antitumour immune response [160, 161]. ITH can be spatial if it occurs at different regions of the tumour and temporal when it is related to clonal evolution [14]. We can currently determine the degree of ITH and characterise each subset of clonal subpopulations [Box 5] based on their specific mutational or transcriptomic profiles. The knowledge of ITH can be of great help in prioritising drug treatments or understanding tumour response to treatment. This section provides an overview of relevant methodologies for the dissection of ITH for guiding drug selection (Fig. 1).

Box 5 **Clonal and subclonal subpopulations**

ITH is characterised by the presence of different tumour subclones, each one of them exhibiting a fitness that daughter cells inherit. Subclones can harbour clonal or trunk mutations, which are present in all cells, and subclonal mutations, which only affect a subset of cancer cells. The prevalence of subclonal alterations can be used to infer a tumour's phylogeny. Treatment can be a source of selective pressure that performs purifying selection on sensitive subclones and increments the fitness of resistant ones.

### 5.1 Genome profiling for targeting tumour clonality

Tumours can harbour clonal mutations, which are present in all cells, and subclonal mutations, which only affect a subset of them. The prevalence of subclonal mutations can be used to infer the tumour's phylogeny, which allows to decipher the order of these mutations and to identify the current subclones and the relationships between them. However, one must take into account that the ability to distinguish between truly clonal and subclonal mutations appearing to be clonal (pseudo‐clonal mutations) depends largely on the number of regions sequenced, the sequencing depth and sample purity [21]. Cancer subclones are subject to Darwinian evolution, and each one of them exhibits a fitness that daughter cells can inherit. Some studies have suggested that increased levels of CNV might be advantageous for the subclone that bears these mutations, which ultimately outcompetes its neighbours [162]. Anticancer drug administration creates a selective pressure that alters subclonal fitness. Drug‐sensitive cells will die, but some subclones, which are usually a minority, may acquire resistance to the treatment and increase their fitness. This resistance might be due to pre‐existent resistant subclones or can arise by *de novo* drug‐induced mutations in drug‐tolerant cells. Eventually, resistant subclones may expand and cause relapse [14]. An interesting example of this behaviour is represented by the stem cell division dynamics described by Xie et al. In this work, they characterised a subgroup of quiescent glioblastoma cancer stem cells (CSC) that evaded antiproliferative chemotherapy and re‐entered the cell cycle, promoting tumour growth and ultimately leading to conventional treatment failure and relapse [163]. Some authors have proposed a combination of multiregion sampling to dissect spatial ITH coupled with monitoring of circulating tumour DNA (ctDNA) via liquid biopsies to measure clonal evolution in real time and adapt the therapy accordingly [164, 165]. Other approaches rely on a Bayesian evolutionary framework to study the spatio‐temporal dynamics of cancer subclones within a single patient [166]. Subclones can be identified using several approaches, including genome profiling and single‐cell sequencing.

Genome profiling is the preferred strategy to study clonal evolution. Several bioinformatics tools have been developed to infer cancer subclones using SNV allele frequencies, CNV profiles and tumour purity measures as input. The most remarkable examples are PyClone‐VI [167], PhyloWGS [168], FastClone [169], SciClone [170] or MOBSTER [171]. However, this approach has several limitations. First, only the mutations that are present in all or the majority of cells will be detected. Moreover, stromal contamination may alter mutation frequencies. Finally, these bioinformatics tools perform many prior inference steps that may introduce errors, which can be propagated in subsequent steps [172].

**Fig. 2:**
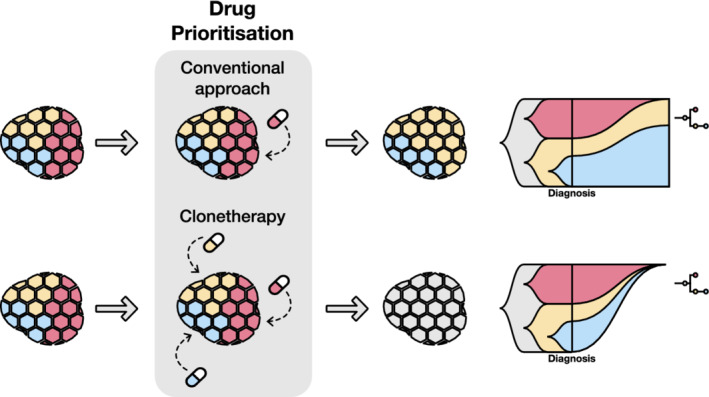
Concept of clonetherapy. The conventional approach in cancer treatment is to target the major subclone, since it is the most represented in a bulk sample. However, if the drug does not hit clonal alterations, other subclones might survive the treatment and expand. Clonetherapy aims to hit both clonal and subclonal alterations identified in a deconvoluted data set in order to target all subclones, thus avoiding potential relapse.

Some drug prioritisation tools initially designed for intertumour heterogeneity have been used for targeting ITH as well [173]. In this work, PanDrugs was run independently for each inferred subclone and the results aggregated in order to prioritise drugs that hit both clonal and subclonal alterations. The term ‘clonetherapy’ was introduced to define the optimal treatment regime that would cover patient ITH by targeting all subclones, including the minority ones with the ability to relapse (Fig. 2).

### 5.2 Single‐cell transcriptomics‐based drug selection

Bulk RNA‐seq allows for the use of the transcriptome as a proxy for elucidating cellular phenotypic traits. This has demonstrated to be useful in uncovering genes important for cancer progression and possible drug targets. However, it involves the averaging of the expression levels in a heterogeneous subpopulation of cells, hiding what might result in important patterns defining tissue dynamics, cell fate and transitions. The idea that the study of predominant cancer subpopulations is insufficient for informing precision oncology has been already suggested in several publications [174, 175]. To prevent or to overcome resistance, we need to implement more accurate molecular profiling techniques. Single‐cell technologies are able to dissect ITH at the scale of individual tumour cells, revealing rare subpopulations and enhancing our understanding of drug resistance and relapse [176, 177]. In this context, the development of single‐cell RNA‐seq (scRNA‐seq) technologies has been seen as a new stepping stone towards an increased understanding of cancer biology.

In recent years, there have been significant advances in the generation of computational tools capable of addressing ITH from a single‐cell point of view. However, the current lack of gold standard analysis guidelines is one of the biggest challenges in the field [178]. Importantly, the generation of community‐maintained and versatile analysis pipelines could help in solving this issue. The bollito pipeline [179], the Web‐Accessible Single Cell RNA‐Seq Processing Platform (WASP) [180] and the Single Cell Interactive Application (SCiAp) [181] are some of the latest efforts in this direction. In general terms, current single‐cell analysis workflows can be subdivided into three main steps: the raw data processing steps or primary analysis; the normalisation and clustering steps, also known as secondary analysis, and the tertiary analysis that involves the functional interpretation of the results. Depending on the sequencing platform, it will be necessary to implement some particularities, although the steps will maintain the same goal (Fig. 1).

The primary analysis is the foundation step in the single‐cell analysis pipeline and refers to the processing of the raw data. It can be subdivided into sample demultiplexing, alignment, QC and quantification. This is a computationally demanding step with the goal of obtaining a matrix of the gene expression profiles for each cell in the experiment. Demultiplexing is usually performed by the sequencer's built‐in software while the most commonly used aligners include Cell Ranger [182] and STARsolo [183]. Pseudo‐aligners such as kallisto or Alevin [184], capable of performing an accurate quantification by mapping the reads directly to the transcriptome, are also frequently used because of their increased speed.

The secondary analysis steps include cell‐based QC, normalisation, dimensionality reduction and clustering of the samples. They can be performed using popular single‐cell analysis toolkits such as Seurat [185] or SCANPY [186]. Nevertheless, it is important to remark that these are not the only methodologies available and that the sparsity (meaning the high fraction of zeroes present in single‐cell matrices) of the analysed data set or the amount of sequenced cells should guide the algorithm selection. Interesting reviews from Duò et al. [187] and Yu et al. [188] could be helpful for performing this algorithm selection. The manifold learning algorithms are recommended for further exploratory single‐cell data visualisation [189]. For instance, the Uniform Manifold Approximation and Projection (UMAP) [190] is considered best practice thanks to its capacity for preserving both global and local structure [178]. The final goal of all these preprocessing steps is to cluster cells based on the identification of distinct biological patterns, cell types and cell states. Here, it is important to note that the selection of a clustering algorithm will strongly impact further downstream analyses [191]. Common clustering methodologies include PhenoGraph [192], SC3 [193] TSCAN [194] or SINCERA [195].

Finally, the tertiary analysis helps to describe and interpret the functional processes that define the biology of each cell subpopulation and thus enable the study of ITH and facilitate finding suitable therapeutic candidates. These downstream steps involve classic DGE methods such as the Wilcoxon rank‐sum test, which has been shown to have an overall robust performance in single‐cell data sets [196, 197] together with bulk‐based methods such as edgeR or limma‐voom. Also, single‐cell specific functional enrichment methodologies such as VISION [198] and UCell [199] apply bulk‐design approaches to individual cells or groups of cells, generating gene signature scores in a similar fashion to bulk methods. Further methodological developments in this context involve prior knowledge‐driven cell‐type annotation using built‐in reference marker collections with SingleR [200] or PanglaoDB [201]. In addition, the study of ligand–receptor interactions between cancer cells and the TME can be crucial for studying the extrinsic factors that contribute to ITH [202] and improving our treatment selections. Tools such as CellPhoneDB [203] or NicheNet [204] are useful for modelling intercellular communication and linking ligands to target genes. Moreover, trajectory inference and expression dynamic methodologies can help us understand both ‘present’ and ‘future’ of the selected subpopulations. While the ‘present’ of a cell is represented by the captured spliced mRNA transcripts, the analysis of the unspliced mRNA can also be used to predict the cell's future transcriptome, the direction and the speed of that change. Slingshot [205] and velocyto [206] or scVelo [207] have been developed to recapitulate the transcriptional dynamics within a data set and furthering our understanding of cell transitions. Such methods are complementary, as Slingshot allows the ordering of cells based on their current snapshot, while velocyto or scVelo facilitates the study of gene regulation by predicting its future steps. Together, all these methodologies facilitate our understanding of the analysed cells, resulting in a better selection of subpopulations of interest and helping in the study of possible clinical targets.

Expression‐based drug prioritisation algorithms are usually applied after functional characterisation of cell subpopulations. Recent methods such as degas associate individual cells with disease attributes such as diagnosis, prognosis and response to therapy [208], whereas Augur prioritises cell types involved in response to perturbations [209]. Beyondcell is a computational method for identifying tumour cell subpopulations with distinct drug responses and proposing cancer‐specific treatments. In order to do this, Beyondcell calculates a drug susceptibility score for each cell, delineates therapeutic clusters defined as groups of cells with a similar drug response and generates a prioritised sensitivity‐based ranking in order to guide drug selection [26].

Still, the lack of information about the spatial context is one the main drawbacks of scRNA‐seq methodologies. This information is of special importance when characterising new subpopulations, since it allows to determine whether the observed differences in expression are a consequence of functional differences or they rely on different interactions with the TME. Additionally, establishing how the TME is going to affect drug tolerance in these subpopulations will be crucial for selecting suitable drug candidates. Spatial transcriptomics (ST) profiling techniques have been recently developed to tackle this question and hold promise of generating much more informed tumoral maps. However, major caveats of this new approach are a lower resolution (still not at the level of single cells) and a lower number of captured genes than scRNA‐seq [210]. In this context, integrative analysis methods for ST are part of a trend aimed at generating a common framework of spatial annotation that will help further enriching scRNA‐seq data sets. Tools such as Tangram [211] or SpaOTsc [212], which map scRNA‐seq data to spatial data collected from the same region, could be used to achieve this goal.

## 6 Incorporating drug prioritisation tools into the clinical practice

Therapy selection guided by bioinformatics approaches is still in its infancy. To date, drug prioritisation methods face technical and biological challenges (Box 6) that constitute clear bottlenecks for their application in routine clinical practice. However, there are currently remarkable efforts to translate these methodologies into medical practices for the benefit of patients.

Box 6 **Main biological and technical challenges associated with the problem of drug prioritisation**

*Biological*

1. Incomplete dissection of inter‐ and intratumour heterogeneity and lack of knowledge of somatic evolutionary processes.
2. Poor understanding of the interface between clonal expansion and cancer initiation.
3. Poor understanding of tumour and TME topological relationships and cell–cell cross‐talking.
4. Exhaustion of antitumor immunity during disease progression.
5. Poor understanding of the specific events leading to the onset and expansion of drug resistant subclones.
6. Incomplete categorisation of short variants, SVs, epigenomic and transcriptional driver alterations and their relationship with drug response.
7. Poor understanding of interrelations between ageing, senescence and drug response.
8. Missing information about the association of germinal variants and ADRs for most anticancer drugs.

*Technical*

1. FFPE preparation of samples, which favours DNA fragmentation, degradation and alterations that are difficult to identify as artefacts during variant calling.
2. Trade‐off between scope and read depth. In genomics, the broader the region sequenced (all regions using WGS, coding‐only using WES or specific genes using targeted sequencing), the lower the coverage. Similarly, the higher the number of sequenced cells, the lower the read depth in single‐cell technologies.
3. Dealing with multi‐alignment reads due to repetitive regions in the genome.
4. Short reads are not sufficient to resolve large SVs, and long‐read sequencing strategies have higher error rates.
5. Lack of gold standard guidelines and information about the spatial context in single‐cell technologies.
6. Predicting toxic interactions or synergistic effects of combination therapies.

The patient journey defines the evolution of cancer patients, describing the different stages from disease prevention to detection, diagnosis, treatment and follow‐up. To diagnose and decide on the best available treatment options, physicians will need integrated patient's information in a clear and interpretable way through clinical decision support systems. Such systems will be able to efficiently access electronic medical records containing multiple data types, including individual genomic data at different points in the patient journey.

**Fig. 3:**
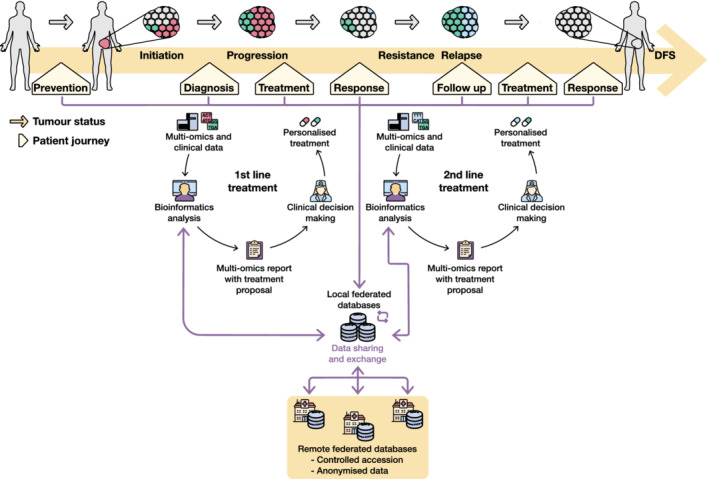
Integration of drug prioritisation methods for clinical decision‐making during the cancer patient journey. Integrated bioinformatics analysis of clinical and multi‐omics data from individual cancer patients would generate a report that includes tumour genomics profiling during the patient journey. Based on such profiles, drug prioritisation methods would provide predictions to propose tailored treatments during the different stages of disease progression. The genomics report would be completed with retrospective treatment response information obtained by comparison with other patients with similar clinical and genomic profiles. Data retrieved at each step of the patient journey would be stored in federated databases for aiding future clinical decisions. DFS, disease‐free survival.

Computational methodologies to analyse and interpret NGS data, including drug prioritisation algorithms, will be incorporated in the clinical decision support systems relying on broad interoperability of data, metadata, research software and computational infrastructure. This will require harmonised nomenclatures, large and well‐annotated genomic data sets linked to patients' clinicopathological information and efficient data exchange (Fig. 3). To address this challenge, multimodal cancer data must be meaningfully connected; thus, data harmonisation and standardisation are crucial. There are several ongoing efforts towards this direction. For instance, the Findable, Accessible, Interoperable, Reusable (FAIR) principles have been proposed to facilitate an efficient clinical data exchange [213]. The NIH Data Commons (https://commonfund.nih.gov/commons) and the Cancer Research Data Commons (CRDC, https://datacommons.cancer.gov/) are further examples of data harmonisation initiatives. On the contrary, initiatives promoting the availability of genomic data linked to enriched clinical annotation have been recently launched such as the ICGC‐ARGO (https://platform.icgc‐argo.org/) [214], which aims to collect a richer data set of cancer genomes with clinical information, health and response to therapy, and the Beyond 1 Million Genomes initiative (B1MG, https://b1mg‐project.eu/), which provides a framework for access and interoperability of genomic and medical data [215]. In addition to computational implementations, the incorporation of multi‐omics approaches and *in silico* drug prioritisation tools into routine clinical practice will require further efforts in the healthcare scenario (Box 7).

Box 7 **Main challenges for the integration of multi‐omics data into routine clinical care**

1. Lack of trained and specialised professionals.
2. Clinical sample accessibility, availability and lack of unified sample processing protocols.
3. Clinical scalability.
4. Lack of standardised gold standard data sets for training and validation of multi‐omics data analysis methods.
5. Deficient computational infrastructures.
6. Implementation of data privacy policies.
7. Implementation of legal and ethical frameworks.

*In silico* drug prioritisation tool performance would also greatly benefit by extensive and standardised clinical, pathological and genomic annotations integrated in a federated data‐sharing model, storing retrospective treatment response information while preserving patients' data privacy. Such a federated data‐sharing framework would also provide benchmarking, training and validation data sets for the evaluation of reliability of novel drug response prediction methods and to identify new predictive biomarkers based on retrospective data [173, 216]. Thus, clinical decision‐making regarding a particular patient would be supported by a genomic report integrating comparative studies of treatment and clinical response obtained from multiple patients with similar genomic profiles. In this sense, the Global Alliance for Genomics and Health (GA4GH) outlines a framework of international policies and standards for the responsible access to genomic and health‐related data [217]. Projects such as the GA4GH Genome Beacons provide a pioneer bioinformatics framework for hospitals to interrogate clinicogenomics data without compromising the privacy and the ownership of the data set [218]. Importantly, such a scenario with controlled accession to clinicogenomics information and secure data sharing would also allow for more robust training, testing and validation of novel drug prioritisation methods, ultimately resulting in direct benefit to patients.

## 7 Conclusions

Cancer is a complex disease that results from the interaction of multiple layers of information. The relationship between tumour origin, the appearance of genomic and transcriptomic variations or microenvironment interactions, all play a role in making tumour treatment challenging. Moreover, cancer is characterised by inter‐ and intratumour heterogeneity, meaning that molecular alterations at multiple levels vary among tumours from different patients, within the same patient or even among cells within the same tumour. For all these reasons, patients may exhibit different responses to the same treatment. As a consequence, there is an urgent need to develop computational methodologies addressing the design of personalised anticancer treatment regimens [173]. Precision oncology aims to address this scenario by proposing patient‐specific treatments tailored to the multi‐omics profiles of individual tumours and the clinical characteristics of each patient. This challenge cannot be met without bioinformatics, since it requires the development, testing and application of algorithms to interpret multi‐source patient data and guide clinical decision‐making. There is an extensive catalogue of drug prioritisation methods pursuing to respond to this demand by proposing tailored treatments based on lists of tumour genetic alterations, gene lists or expression profiles.

This article has reviewed the state of the art in computational drug selection methodologies. It also reviewed the bioinformatics methods currently available for the processing, analysis and interpretation of genomics and transcriptomics data. In particular, the computational approaches used for the dissection, characterisation and drug prioritisation for the therapeutic management of ITH, a major cause of variability in responses to cancer treatment, were also described.

Overall, these computational drug prioritisation methods still rely on the one target–one drug–one disease notion, in contrast to current therapeutic approaches, which often combine a rational and drug‐based synergistic therapeutic regime [219]. Moreover, cancer treatment research has shifted from a cancer‐centred model to an TME‐centred model [220] and there are still a few methodologies oriented in this direction. Some bioinformatics efforts predict drug combination therapies [221] or suggest TME drug immunomodulators [142] based on omics profiles, but to date, very few methods exist as these areas are underexplored and the challenge remains unsolved. Bioinformatics is crucial to meet the goal of designing precision medicine‐based therapies [222] being capable of selecting tailored treatments targeting tumour heterogeneity efficiently and playing a key role in its incorporation into the clinical practice.

## Conflict of interest

The authors declare no conflict of interest.

## Author contributions

MJJ‐S and TDD wrote the short variants and structural variants sections. MJJ‐S and SG‐M described the multi‐omics section. MJJ‐S also wrote the tumour clonality subsection. SG‐M wrote about the transcriptomics‐based drug selection. CF‐T wrote about the single‐cell RNA‐seq subsection. MJJ‐S, GG‐L and FA‐S wrote the outline and contributed to all sections. MJJ‐S, GG‐L and FA‐S conceptualised the figures. MJJ‐S created the figures. MJJ‐S, SG‐M and CF‐T wrote the tables. All authors read and approved the final manuscript.
